# *Plectranthus amboinicus* essential oil and carvacrol bioactive against planktonic and biofilm of oxacillin- and vancomycin-resistant *Staphylococcus aureus*

**DOI:** 10.1186/s12906-017-1968-9

**Published:** 2017-09-16

**Authors:** Sara Edwirgens Costa Benício Vasconcelos, Hider Machado Melo, Theodora Thays Arruda Cavalcante, Francisco Eduardo Aragão Catunda Júnior, Mário Geraldo de Carvalho, Francisca Gleire Rodrigues Menezes, Oscarina Viana de Sousa, Renata Albuquerque Costa

**Affiliations:** 1 Master’s Program in Biotechnology, INTA College, Sobral, 62050-100 Brazil; 2 State University of the Tocantina Region of Maranhão, Imperatriz, MA 65901-480 Brazil; 3 Chemistry Department, Federal Rural University of Rio de Janeiro, Seropédica-RJ, Rio de Janeiro, 23890-000 Brazil; 4 Sea Sciences Institute, Federal University of Ceará, Fortaleza, CE 60165-081 Brazil

**Keywords:** *Staphylococcus aureus*, Drug-resistant bacteria, *Plectranthus amboinicus*

## Abstract

**Background:**

The emergence of multidrug-resistant bacteria is a worldwide concern and in order to find an alternative to this problem, the occurrence of antimicrobial compounds in *Plectranthus amboinicus* essential oil was investigated. Thus, this study aims to determine susceptibility of *Staphylococcus aureus* isolated from food to antibiotics, *P. amboinicus* essential oil (PAEO) and carvacrol.

**Methods:**

Leaves and stem of *P. amboinicus* were used for extraction of essential oil (PAEO) by hydrodistillation technique and EO chemical analysis was performed by gas chromatography coupled to a mass spectrometer. *S. aureus* strains (*n* = 35) isolated from food and *S. aureus* ATCC 6538 were used to evaluate the antimicrobial and antibiofilm activity of PAEO and carvacrol. All strains (*n* = 35) were submitted to antimicrobial susceptibility profile by disk diffusion method. Determination of MIC and MBC was performed by microdilution technique and antibiofilm activity was determined by microtiter-plate technique with crystal violet assay and counting viable cells in Colony Forming Units (CFU).

**Results:**

Carvacrol (88.17%) was the major component in the PAEO. Antibiotic resistance was detected in 28 *S. aureus* strains (80%) and 12 strains (34.3%) were oxacillin and vancomycin-resistant (OVRSA). From the 28 resistant strains, 7 (25%) showed resistance plasmid of 12,000 bp. All strains (*n* = 35) were sensitive to PAEO and carvacrol, with inhibition zones ranging from 16 to 38 mm and 23 to 42 mm, respectively. The lowest MIC (0.25 mg mL^−1^) and MBC (0.5 mg mL^−1^) values were observed when carvacrol was used against OVRSA. When a 0.5 mg mL^−1^ concentration of PAEO and carvacrol was used, no viable cells were found on *S. aureus* biofilm.

**Conclusion:**

The antibacterial effect of carvacrol and PAEO proves to be a possible alternative against planktonic forms and staphylococcal biofilm.

## Background

*Staphylococcus aureus* is a Gram-positive cocci often associated with gastroenteritis acquired from contaminated foods such as milk [1] and shrimp. The occurrence of multidrug-resistant bacteria in food is a worldwide concern [2], and *S. aureus* drug-resistant to beta-lactams has been isolated from dairy products [1] and shrimp [3].

Some factors act for selecting drug-resistant bacteria pathogens, such as inappropriate use of antibiotics, and/or the inadequate disposal of antimicrobial drugs in the environment [4, 5]. Thus, the inappropriate use of antibiotics in aquaculture [6, 7] and livestock [8] has contributed to the selection of resistant microbial species, and, consequently, increased food contamination level of animal origin and derivatives.

As an alternative to mitigate the occurrence of multidrug-resistant bacteria, the study of antimicrobial compounds in phanerogams has been proposed [9]. In this context, the plants of genus *Plectranthus* - 3000 recognized species, spread along countries in Africa, South America, Asia and Australia – are widely recognized in folk and popular medicine, being employed in digestive treatments as well as in infectious, inflammatory and respiratory problems [10, 11]. Species of *Plectrantus* [12, 13], including *P. amboinicus* [14], has been studied due to its pharmacological properties in order to validate its popular use.

Bioactivity of *P. amboinicus* is related to the occurrence of 76 volatiles and 30 non-volatile compounds belonging to different classes of phytochemicals (monoterpenoids, diterpenoids, triterpenoids, sesquiterpenoids, phenolics, flavonoids, esters, alcohols and aldehydes) [15]. Studies about the pharmacological activities of *P. amboinicus* are conducted from extracts or essential oil, i.e., complex volatile compounds, synthesized naturally in different plant parts during the process of secondary metabolism with great potential in the field of biomedicine [16]. In this sense, *P. amboinicus* crude essential oils could be used as a tool for the developing novel and more efficacious antimicrobial agents against *S. epidermidis* and *Candida* species [17]*.*

The susceptibility of methicillin-resistant *S. aureus* (MRSA) clinical isolates to extracts of *P. amboinicus* has been reported [18], however, studies about the action of this oil and its major component against planktonic and biofilm of drug-resistant *S. aureus* from food are still incipient.

Thus, this study aims to determine the susceptibility of drug-resistant *S. aureus* strains isolated from milk and shrimp to *P. amboinicus* essential oil (PAEO) and its major component - carvacrol.

## Methods

### Strains origin

Thirty five *Staphylococcus aureus* strains isolated from pasteurized milk (*n* = 12) and shrimp (*n* = 23) were used. All strains are part of the bacterial catalogue available at Center for Applied Molecular Bioprospecting and Experimentation (NUBEM - INTA College) and had the following biochemical characteristics: Gram-positive cocci, catalase-positive, manitol-positive and coagulase-positive. The strains, maintained in Tryptone Soy Broth with 20% glycerol, were plated onto Mannitol Salt Agar (35 °C for 48 h) and isolated Tryptone Soy Agar for performing microbiological analyzes.

### Antibiogram

As screening for the selection of drug-resistant strain for Minimum Inhibitory Concentration (MIC) and minimum bactericidal concentration (MBC) and antibiofilm activity, all strains (*n* = 35) were submitted to antimicrobial susceptibility profile by disk diffusion method using Mueller Hinton Agar [16]. The following antibiotics discs were used: oxacillin (1 μg), penicillin G (10 U), ampicillin (10 μg), cefotaxime (30 μg), ceftriaxone (30 μg), cefepime (30 μg), imipenem (10 μg), vancomycin (30 μg), gentamicin (10 μg), and chloramphenicol (30 μg). Antimicrobial susceptibility was detected by analyzing the inhibition zones, measured and compared according the standards set by the CLSI (2012) [19] which classify the strains as sensitive, intermediate or resistant.

### Plasmid DNA extraction

*S. aureus* resistant strains were inoculated in Brain Heart Infusion broth and cultured for 24 h at 35 °C. Then, 2.0 mL of each culture was retrieved for plasmid DNA extraction with a commercially available extraction kit (GeneJET Plasmid Miniprep Kit). A 3.0 μL aliquot of each sample of extracted DNA was submitted to 1.5% agarose gel electrophoresis in TBE buffer for 60 min at 120 V, 200 mA and 100 w. The plasmid DNA was viewed under UV light with a Spectrolinetransilluminator and photographed with a Kodak EDAS 290.

### Plant material

Leaves and stem of *P. amboinicus* were collected in October 2014, on a farm (3.49′52,16″S, 40.24′38,38″W) located in Cariré (Northest Brazil). The botanical identification was made at Herbarium Professor Francisco José de Abreu Matos – (HUVA), Acaraú Valley State University (Sobral-CE). The dried specimens were deposited in the HUVA and registered under specific numbering 18,416.

### Extraction of *Plectranthus amboinicus* essential oil (PAEO)

Extraction of PAEO was made at the NUBEM (INTA College), using the hydrodistillation technique. Four extractions were performed, using 7.690 Kg of plant in order to obtain 5.2 mL essential oil, approximately. PAEO was transferred to sterile amber vial, which was covered with aluminum foil and maintained at 2 to 8 °C. An aliquot of approximately 20 μL was used for chemical analysis.

### Chemical analysis of the essential oil (PAEO)

The essential oil chemical analysis was performed by gas chromatography coupled to a mass spectrometer (GC-MS) using a gas chromatograph (Shimadzu QP2010 Plus), and helium (He) as carrier gas. A capillary column Factor Four/ VF-5 ms, 30 m length, 0.25 mm internal diameter, 0.25 mm film thickness was used. The carrier gas flow rate was 1 mL min^−1^. The initial oven temperature was 60 °C. After heating for 2 min, it increased at a constant rate of 2 °C per minute up to 110 °C; then 3 °C per minute to 150 °C; and 15 °C per minute to 290 °C to a final, isotherm 290 °C for 17 min. The injector and detector temperatures were respectively 250 °C and 310 °C. The mode of injection was split and the injection volume was 1 mL. Mass spectra were produced by electron impact (70 eV). Quantitative analysis of the essential oil composition was made on a gas chromatograph, coupled with HP5890 Series II ionization detector, using the same operating conditions and the same type of column as the CG/EM analysis (except for the injector and detector temperatures, which were of 220 °C and 250 °C, respectively). The percentage of each constituent is calculated by the integral of the area under the respective peaks to the total area of all the constituents of the sample. The various constituents of the essential oil were identified by visual comparison of their mass spectra with those in the literature [20] and with actual standards in Nist08 library computer system, as well as by comparing retention rates with those in the literature [20]. A standard solution of n-alkanes (C8-C20) was injected under the same chromatographic conditions used to provide samples and to get retention rates.

### PAEO and carvacrol antimicrobial effect

Antimicrobial potential of OEPA and carvacrol was determined by: (1) antibiogram, (2) determination of minimum inhibitory concentration (MIC), (3) minimum bactericidal concentration (CBM) and (4) antibiofilm activity.

### Antibiogram to PAEO and carvacrol

The antimicrobial activity of PAEO and its major component was performed from the disk diffusion test on Mueller Hinton Agar, as detailed on the Antibiogram item. Sterile paper discs (6 mm in diameter) were soaked with PAEO (20 μL) and 98% Carvacrol (20 μL) (Sigma). The antimicrobial effect was detected by the formation of inhibition zones around the disks soaked with PAEO and carvacrol. The halos were measured in millimeters, labeled as bioactive those larger than 15 mm. As positive and negative control, tetracycline (30 μg) and disks soaked with 20 μLTween 80 solution 3% were used, respectively.

### Minimum inhibitory concentration (MIC) and minimum bactericidal concentration (MBC)

Determination of MIC and MBC was performed by microdilution technique in Tryptone Soy Broth (TSB) [19], using polystyrene plates of 96 wells and two strains: *S. aureus* ATCC 6538, and oxacillin- and vancomycin-resistant *S. aureus* (OVRSA).Concentration of both strains was adjusted in TSB to 1.25 × 10^7^ Colony Forming Units (CFU mL^−1^).20 replicates of each strain were tested with PAEO and carvacrol in concentrations of 4 mg mL^−1^, 2 mg mL^−1^, 1 mg mL^−1^, 0.5 mg mL^−1^, 0.25 mg mL^−1^, 0.125 mg mL^−1^ and 0.0625 mg mL^−1^. MIC was determined from visual reading and was considered the concentration able to inhibit microbial growth. In order to determine the MBC, a pool was made of 10 wells for each concentration, and then plating was performed in 10 μL in triplicate Tryptone Soy Agar. The concentration which was not verified microbial growth was considered CBM.

### Antibiofilm activity

Antibiofilm activity was determined by microtiter-plate technique [21] with crystal violet (CV) assay, counting viable cells in Colony Forming Units (CFU) [22]. In order to determine the PAEO and carvacrol action in the biofilm, plates were subjected to optical density microplate reading (Molecular Devices -SpectraMaxParadigmMulti-Mode) at a wavelength of 570 nm. Activity was found in concentrations that failed to display the adhesion of crystal violet in any of the wells.

### Controls

For CIM, CBM and antibiofilm activity, TSB containing the test substance (PAEO or carvacrol) in concentrations of 4 mg mL^−1^, 2 mg mL^−1^, 1 mg mL ^−1^, 0.5 mg mL ^-1^, 0.25 mg mL ^-1^, 0.125 mg mL^−1^ and 0.0625 mg mL^−1^ was used as turbidity control. For contamination control, only the culture medium (TSB) in three wells on the plates we used. Negative control was done by inoculating 100 μL of strain (1.25 × 10^7^ CFU mL^−1^) in wells containing culture medium (TSB). As positive control, tetracycline in the same concentration of test substances was used: 4 mg mL^−1^, 2 mg mL^−1^, 1 mg mL ^−1^, 0.5 mg mL ^-1^, 0.25 mg mL ^-1^, 0.125 mg mL^−1^ and 0.0625 mg mL^−1^.

### Statistical analysis

Data analysis in GraphPad Prism 5 with ANOVA statistical, followed by a Student-Newman-Keuls test for significant variability comparisons (*p* < 0.001) between different concentrationswere used to conduct the analysis.

## Results

**Fig. 1:**
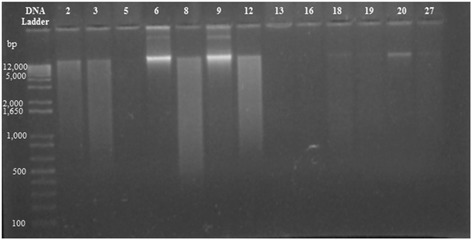
DNA Plasmid electrophoresed on 1.5% agarose gel. Lane 1: DNA Ladder. DNA Plasmid 12,000 bp instrains2 (Lane 2), 3 (Lane 3), 6 (Lane 5), 8 (Lane 6), 9 (Lane 7), 12 (Lane 8) and 20 (Lane 13)

Antibiotic resistance was observed in 28 *S. aureus* strains (80%) and ten resistance profiles were observed: three profiles of monoresistance (*n* = 5, 17.8%), three cross-resistance to beta-lactams (*n* = 8; 28.6%) and four multidrug resistant (*n* = 15; 53.6%) (Table 1). From the 28 resistant strains, 7 (25%) showed plasmid of 12,000 bp (Fig. 1).

**Table 1:** Antimicrobial resistance profile of 28 *Stapylococcus aureus* strains isolated from milk and shrimp

Profile	Drugs	N° of Isolates		Total (%)
		Shrimp	Milk	
Monoresistance	Pen	1	0	5 (17.8)
	Oxa	3	0	
	Cro	1	0	
Cross-resistanceto beta-lactam	Pen + Amp	2	0	8 (28.6)
	Oxa + Amp	3	0	
	Pen + Amp + Oxa + Cro + Ctx + Cpm	3	0	
Multidrug resistance	Amp + Van	1	0	15 (53.6)
	Pen + Amp + Gen	1	0	
	Pen + Amp + Oxa + Van	1	11	
	Pen + Amp + Oxa + Ctx + Van	0	1	
	Total	16	12	28 (100)

*PEN* penicillin G, *OXA* oxacillin, *CRO* Ceftriaxone, *AMP* ampicillin, *CTX* cefotaxime, *CPM* cefepime, *VAN* vancomycin, *GEN* gentamicin

**Fig. 2:**
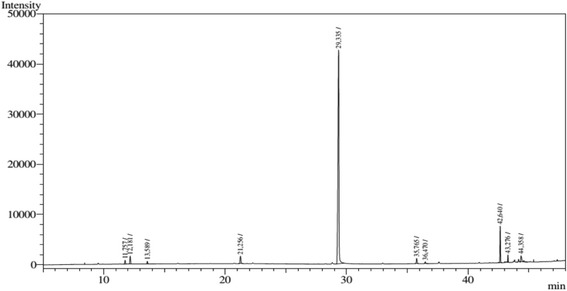
CG-MS chromatogram analysis of *Plectranthus amboinicus* essential oil

Carvacrol (88.17%) was the major component in the essential oil of *P. amboinicus,* followed by caryophyllene oxide, 1,8-cineole, 4-terpinenol, alpha-cisbergamoteno, p-cymene and gamma- terpinene (Table 2, Fig. 2).

**Table 2:** Chemical characterization of the essential oil extracted from leaves of *Plectranthus amboinicus*

Components	CRI	RI_LIT_	RT_CG/FID_	%
p-cymene	1029	1026	11.757	0.72
1,8-cineol	1037	1031	12.181	2.01
Gamma-terpinene	1063	1059	13.589	0.45
4-terpineol	1184	1177	21.256	1.78
Carvacrol	1303	1299	29.335	88.17
Alpha-cisbergamoteno	1420	1412	35.765	0.84
NI	1435	–	36.470	0.17
Caryophyllene oxide	1591	1583	42.640	5.85
Total				99.99%

*CRI* calculated retention index, *RIlit* retention index of the literature, *RT* retention time of CG-FID, *NI* unidentified

All strains (*n* = 35) of *S. aureus* were sensitive to PAEO and carvacrol with inhibition zones ranging from 16 to 38 mm and 23 to 42 mm, respectively (Table 3). The standard strain of *S. aureus* ATCC 6538 showed a inhibitory halo of 34 and 47, respectively, for PAEO and carvacrol.

**Table 3:** Distribution of the *Staphylococcus aureus* strains according to inhibition zone size against the essential oil *Plectranhus amboinicus*, carvacrol and tetracycline

Inhibition halos interval (mm)	Shrimp (*n* = 23)			Milk (*n* = 12)		
	PAEO	Carvacrol	TCY	PAEO	Carvacrol	TCY
16–20	0	0	1	4	0	1
21–25	4	2	3	3	1	11
26–30	16	12	6	5	3	0
31–35	2	4	12	0	8	0
36–40	1	3	1	0	0	0
41–42	0	2	0	0	0	0

*PAEO Plectranthus amboinicus* essential oil. *TCY* tetracycline 30 μg. *n* number of bacterial strains. *mm* millimeters

MIC value of 0.25 mg mL^−1^ was observed when PAEO were used against *S. aureus* ATCC 6538 and carvacrol was used against *S. aureus* ATCC 6538 and OVRSA. The lowest MBC value (0.5 mg mL^−1^) was detected when carvacrol was used against OVRSA (Table 4).

**Table 4:** Minimum Inhibitory Concentration (MIC mg mL^−1^) and Minimum Bactericidal Concentration (MBC mg mL^−1^) of *Plectranthus amboinicus* essential oil and carvacrol

Substances	Activity antimicrobial	*Staphylococcus aureus*	
		ATCC 6538	OVRSA
PAEO	MIC	0.25	0.5
	MBC	2	1
Carvacrol	MIC	0.25	0.25
	MBC	1	0.5

*ATCC* American Type Culture Collection, *OVRSA* Oxacillin and vancomycin-resistant *Staphylococcus aureus*

**Fig. 3:**
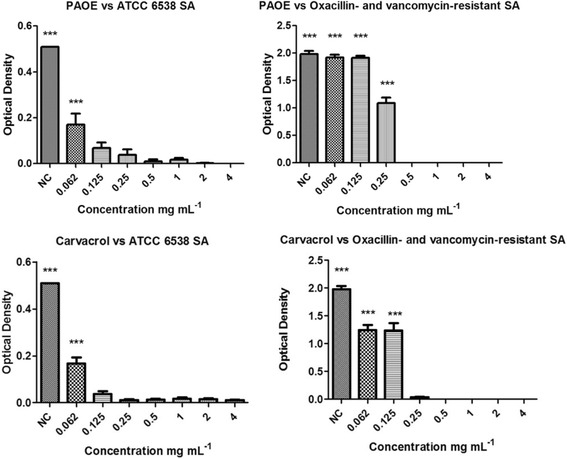
Optical density of bacterial biofilm in *S. aureus* ATCC 6538 and *S. aureus* isolated from food, treated with OEPA and carvacrol in varying concentrations (0.062, 0.125, 0.25, 0.5, 1, 2 and 4 mg mL^−1^). ANOVA, followed by Student-Newman-Keulstest ***p < 0.001 vs. all variables.*SA: *Staphylococcus aureus*. NC: negative control

Biofilm activities against *S. aureus* ATCC 6538 and OVRSA were observed in concentrations of 0.5 mg mL^−1^ (OEPA) and 0.25 mg mL^−1^ (carvacrol) (Fig. 3). However, inhibition potential was detected in all tested concentrations of carvacrol and OEPA, in comparison to the negative control. Tetracycline inhibits biofilm formation (*S. aureus* ATCC 6538 and OVRSA) in all concentrations tested (0.062, 0.125, 0.25, 0.5, 1, 2 and 4 mg mL^−1^).

## Discussion

Multidrug-resistant profile was detected in 15 strains (53.6%) (Table 1). Some studies have described the incidence of multidrug-resistant strains of *S. aureus* in foods and in those who handle them, confirming the assertion that one of the main sources of food contamination by *S. aureus* is the handler [23, 24]. Contamination of foods by *S. aureus* multiresistant are related to care in handling, processing and transport of food, as well as correlation of bacterial resistance to the indiscriminate use of antimicrobials in agriculture and aquaculture [2, 25].

In addition to the considerable rate of penicillin resistance, it is valuable to mention the high incidence of strains resistant to oxacillin (*n* = 22; 78.6%) (Table 1). Oxacillin resistance is associated of the *mec*A gene, which encodes a protein with low binding affinity to β-lactam. Thus, in vitro resistance to oxacillin indicated resistance to all other β-lactam drugs [26, 27].

In case of infection caused by strains resistant to oxacillin, the clinically chosen therapy is vancomycin. Thus, another fact worth mentioning in this study is that 14 (50%) of 28 all the resistant strains would persist against vancomycin (Table 1). The present research corroborate studies of Akindolire et al. (2015) [28] and Nunes et al. (2016) [29], that reporting a high incidence (60–100%) of *S. aureus* strains resistant to vancomycin in isolated samples of milk or its derivatives. Baumgartner et al. (2014) [23] reported the presence of *S. aureus* carriers of beta-lactam resistance genes, methicillin and vancomycin in foods, including dairy products.

Of the 28 resistant strains, 7 (25%) showed plasmid of 12,000 bp (Fig. 1) that are probably related to resistance, since these seven strains were isolated from milk and presented multi-drug resistance profile (Pen + Amp + Oxa + Van). For McCarthy and Lindsay (2012) [30], plasmids are responsible for the spread of virulence and resistance genes in *S. aureus* populations. The authors assigned a total of 39 plasmid groups of *Staphylococcus aureus* (p*GSA*) based on the combination of the *rep* genes each plasmid had and revealed 28 plasmid groups with large genomes (>15Kb),which carried a diverse range of genes.

Our findings corroborate several studies when reporting carvacrol as a major component of *Plectranthus amboinicus* essential oil. On the other hand, the concentration of carvacrol (88.17%) in the PAEO obtained in the present study (Table 2) was greater than the variation already described in literature - from 28 to 70%. Compounds such as γ-terpinene, ρ-cement and caryophyllene oxide have been found in smaller amounts when compared to data from this study [17, 31, 32]. Phytocompounds variation in essential oils may be related to the time of botanical material collection, region, soil and seasons [33]. Although detected in small quantities (Table 2), it is known that p-cymene boosts the pharmacological effects of carvacrol [34].

All strains (*n* = 35) of *S. aureus* were sensitive to PAEO and carvacrol. Most strains isolated from milk showed sensitivity to tetracycline with halos of 21–25 mm (*n* = 11; 91.6%) (Table 3). When subjected to the test with carvacrol, 100% (*n* = 12) demonstrated halos size of an equal sensitivity or greater than those of positive control. Moreover, inhibition halos of an equal or greater than those of positive control were observed in 66.6% of the tested strains from milk with OEPA.*S. aureus* strains isolated from shrimp showed sensitivity to tetracycline and most of them presented halos between 31 to 40 mm (*n* = 13, 56.52%) in size (Table 3). When tested with carvacrol and OEPA, 39.13% (*n* = 9) and 13.04% (n = 3) strains showed, respectively, halos sizes greater than or equal to control. Thus, the carvacrol was also more effective when used against the strains of shrimp samples. In this study, the antimicrobial activity of PAEO seems to be related to the concentration of carvacrol (88.17%), whereas all strains were susceptible to both compounds tested and the largest halos were observed when using carvacrol.

Providing support to the findings of this research, Ajitha et al. (2014) [35] found potential inhibition of the species *P. amboinicus* against *Staphylococcus* spp., *Escherichia coli*, *Pseudomonas* spp. and *Bacillus* spp. The authors describe halos measuring around 10 mm, i.e., values lower than those found in the present study.

Rodrigues et al. (2013) [36] evaluated the antimicrobial activity of OEPA containing 67.9% carvacrol, and found that the MIC against *S. aureus* ATCC 12692 (0.128 mg mL^−1^) was higher than MIC against the strain of multidrug-resistant *S .aureus* (0.032 mg mL^−1^). In the present study, it was observed that the MIC for OEPA OVRSA strain (0.5 mg mL^−1^) was higher than the MIC for *S. aureus* ATCC 6538 (0.25 mg mL^−1^). The difference between the two studies may be related to the chemical composition of the oils, since the OEPA obtained by Rodrigues et al., (2013) [36] had 13 compounds including thymol (1.3%) and thymol methyl ether (1.6%), while the OEPA used inthe present study contained seven constituents, with a concentration of carvacrol (88.17%) higher.

Regarding the CIM carvacrol, the same value (0.5 mg mL^−1^) was observed when both strains (*S. aureus* ATCC 6538 and OVRSA) were tested (Table 4). Carvacrol and thymol present the ability to dissolve themselves in the cytoplasmic membrane, aligning the fatty acid chains and providing an increase in the passive permeability of the membrane.

Considering the values of CBM, OEPA and carvacrol showed better results against the OVRSA strain (Table 4). This finding is of utmost importance, since it demonstrates a promising potential of those two bioactive substances as antimicrobial agents tested against medically resistant strain. Hosseinkhani et al. (2016) [37] also obtained promising results when testing the antibacterial activity of an essential oil rich in monoterpenes against multidrug resistant strains of *S. aureus*. According to the authors, the results may indicate the possibility of using monoterpenes incorporated into antimicrobial formulations in order to treat infections caused by multi-resistant bacteria.

In the present study, the isolated carvacrol demonstrated a bactericidal activity against both strains (ATCC and OVRSA) twice as effective when compared to OEPA. This fact may be explained considering that biological action of essential oils is probably related to the synergy between all its compounds, and not only to the action of their main constituent [38]. Sokovic et al. (2010) [39] tested several essential oils with therapeutic potential and also noted that the carvacrol compound had higher activity against *S. aureus* when compared with essential oils.

Souza et al. (2013) [40] investigated the influence of carvacrol on *S. aureus* strains isolated from food, and the monoterpenestrongly interfered in the permeability of the plasmic membrane, in halotolerance, in coagulase activities, and in the production of enterotoxins. In addition, the authors report that the damage caused by carvacrol in the membrane is accompanied by important changes in the surface cells of *S. aureus*.

Figure 3 shows the biofilm reduction of two strains of *S. aureus* caused by the use of OEPA and carvacrol. All OEPA and carvacrol concentrations had inhibitory effect on the biofilm of ATCC strains, as a statistically significant difference (*p* < 0.001) was observed when compared to the negative control. Regarding the OVRSA strain, all carvacrol concentrations showed an inhibitory effect on the biofilm, but complete inhibition was detected at a concentration of 0.25 mg mL^−1^. The OEPA had inhibitory effect against biofilm OVRSAwhen used concentration of 0.25 mg mL^−1^, but only completely inhibited biofilm at a concentration of 0.5 mg mL^−1^.Studies often relate the effect of inhibiting biofilm formation OEPA and carvacrol to: (1) damage to the cell envelope, leading to cell death and to the ability to attach biofilms; and (2) a reduction in the expression of quorum sensing activation of genes, due to decreased bacterial density caused by a delay in the development of cell growth in planktonic cells [41, 42]. Yadav et al. (2015) [43] found activity of carvacrol in combination with eugenol against against Methicillin-Resistant and Methicillin-Sensitive *Staphylococcus aureus* biolfim.

Arfa et al. (2006) [44] relate antibiofilm activity of carvacrol with the presence of free hydroxyl group, as carvacrol without this composition does not show antibacterial activity. In addition, the mixture of free hydroxyl with other chemical compounds enhances the effect against biofilm carvacrol [45]. Although its mechanism is not fully understood, evidences indicate that its antimicrobial activity is due to: (1) Increase the permeability of the cytoplasmic membrane, silted H^+^ into the cell by binding to the hydroxyl group and (2) modification of fatty membrane acids [34].

## Conclusions

Our results show the antibacterial and antibiofilm potential of PAEO and carvacrol against the *S. aureus* standard strain and, especially, against oxacillin- and vancomycin-resistant *S. aureus*. Considering that *P. amboinicus* is a plant used in popular medicine practices in Brazil, more studies on the in vivo antibacterial and toxicity are recommended.
